# Quantitative Analysis of Lactobionic Acid in Bioreactor Cultures and Selected Biological Activities

**DOI:** 10.3390/molecules29225400

**Published:** 2024-11-15

**Authors:** Kamila Goderska, Wojciech Juzwa, Tomasz M. Karpiński

**Affiliations:** 1 Department of Food Technology of Plant Origin, Faculty of Food Science and Nutrition, Poznan University of Life Sciences, Wojska Polskiego 31, 60-624 Poznan, Poland; 2 Department of Biotechnology and Food Microbiology, Faculty of Food Science and Nutrition, Poznan University of Life Sciences, Wojska Polskiego 48, 60-627 Poznan, Poland; wojciech.juzwa@up.poznan.pl; 3 Chair and Department of Medical Microbiology, Poznań University of Medical Sciences, Rokietnicka 10, 60-806 Poznan, Poland; tkarpin@ump.edu.pl

**Keywords:** lactobionic acid, *Pseudomonas taetrolens*, whey, lactose, flow cytometry, *Bifidobacterium*, prebiotic, in vitro model

## Abstract

The aim of this study was to quantitatively analyse lactobionic acid obtained from bioreactor cultures using whey as a liquid medium with bacteria of the *Pseudomonas taetrolens* species. The most important culture parameters affecting the production of the acid are indicated and evaluated. The highest lactobionic acid yield was 37.42 g/L, selecting the appropriate strain (*Pseudomonas taetrolens* 4′) and environmental conditions (2% lactose concentration in the bioreactor). The amount of lactose and lactobionic acid was determined by high-performance liquid chromatography. Microorganism analysis was also carried out using a flow cytometer with imaging to study the metabolic activity of microorganisms during lactobionic acid production. In addition, during the study, Bifidobacteria were microencapsulated with lactobionic acid and their survival was evaluated in an in vitro model of the gastrointestinal tract, checking the prebiotic properties of the acid. The highest number of viable cells in the microcapsules after digestion was obtained using the *Bifidobacterium bifidum* strain DSM 20082. The antagonistic activity of lactobionic acid was also analysed.

## 1. Introduction

Lactobionic acid (LBA) belongs to the group of bionic acids and consists of a galactose molecule linked to a gluconic acid molecule. It has found application in many areas of industry—such as medicine, pharmaceuticals and cosmetics—due to its moisturising, chelating, antioxidant, preservative and regenerative properties. For several years, it has been successfully used in the food industry, where it fulfils its role as a calcium carrier and water retaining agent in food products. It is also distinguished by its acidifying and antimicrobial properties. Lactobionic acid also demonstrates its prebiotic effects—it is poorly absorbed at the small intestinal stage and remains stable against the action of digestive enzymes. Lactobionic acid is obtained by oxidation of lactose. On an industrial scale, the acid is obtained by chemical oxidation, which is expensive and harmful to the environment. The solution is to produce lactobionic acid by microbial means through enzymatic oxidation of lactose using microorganisms of the genus *Pseudomonas* sp. Numerous studies confirm that the production of lactobionic acid with the bacteria *Pseudomonas taetrolens* results in high yields. Increasingly, whey, a difficult by-product of the dairy industry, is promisingly being used as a source of lactose in microbial acid production. Thus, the microbial production of lactobionic acid by *Pseudomonas taetrolens* using whey as a lactose source, with appropriate environmental conditions (pH, temperature, aeration, inoculum concentration) may prove to be a non-invasive, cost-effective method for use in food technology.

### 1.1. Lactose Oxidation with Microorganisms

Lactobionic acid (4-O-β-galactopyranosyl-D-gluconic acid) (LBA) is an aldonic acid, a product of lactose oxidation, containing gluconic acid and galactose, and is characterised by its unique moisturising activity. Other physicochemical properties such as antioxidant, antimicrobial and metal chelating activities have provided it with potential uses in the medical, pharmaceutical and cosmetic industries. Furthermore, LBA is biodegradable, incompatible and non-toxic. As a low-calorie, gelling, humectant and texturizing substance, it has gained attention as a food additive. In addition, it is used as a prebiotic [1,2].

On an industrial scale, lactobionic acid is mainly produced by a chemical method. However, this process is energy-intensive, costly and environmentally harmful due to the reaction catalysts and process residues. In recent years, the production of LBA by microbial means has become increasingly popular due to its non-toxicity and easier reaction process. Researchers’ attention has focused on production via enzymatic catalysis and microbial bioconversion. However, it should be noted that enzymes can be unstable in industrial environments, complicated to prepare and require cofactors for activation. Microbial cells are more robust, easier to prepare and have lower production costs. Many bacteria and fungi show the ability to oxidise lactose to lactobionic acid. The most important microorganisms include bacteria from the genera *Pseudomonas, Acetobacter* and *Zymomonas* [3].

Bacteria of the genus *Pseudomonas* produce lactobionic acid via the lactose oxidation pathway. A dehydrogenase catalyses the oxidation of lactose to lactobion-δ-lactone using flavinadenine dinucleotide as an electron transfer system. The lactone is then hydrolysed by lactonase to lactobionic acid. This process is usually carried out at 25–50 °C at pH 6. The end product of the reaction can be lactobionic acid or its salts. If lactobionate salts are obtained, the mixture is passed through purification methods to obtain pure lactobionic acid. The production of LBA is significantly influenced by environmental factors. Among the most important are temperature, pH, aeration, inoculation level of the inoculum and the culture stage of the inoculum used [4].

### 1.2. Characteristics of Pseudomonas Taetrolens

The production of lactobionic acid using whey by microbial means has become a cost-effective and advantageous alternative to its industrial production by chemical means. LBA is a polyhydroxy acid produced by *Pseudomonas taetrolens* during the fermentation process. This process can be disrupted by the excessive supply of dissolved oxygen, inadequate pH control measures, metabolic overload and stress-induced physiological reactions during the process. The substrate versatility of *Pseudomonas taetrolens* in a co-digestion system combining cheese whey with glucose, glycerol or lactose as a co-substrate was investigated. The presence of glycerol increased cell density, but reduced fermentation efficiency. In contrast, the supply of lactose stimulated the overproduction of lactobionic acid with high volumetric efficiency. Glucose supply, on the other hand, led to simultaneous production of lactobionic acid and gluconic acid. The proven substrate versatility of *Pseudomonas taetrolens* can be used to co-produce two valuable organic acids and to produce lactobionic acid in a highly efficient and effective manner [5].

*Pseudomonas taetrolens* shows a high bioconversion capacity per unit mass, without complex nutritional requirements. Due to its unique functional characteristics and the possibility to use whey as a fermentation substrate, *P. taetrolens* has become a robust matrix for the industrial bioproduction of lactobionic acid. An important feature is that lactobionic acid production occurs mainly after cell growth has stopped. By decoupling the phase of metabolite formation from cell growth, it is possible to upgrade the cell’s resources and ensure a balanced metabolic state. This effect is particularly useful in increasing cell performance. The separation of lactobionic acid formation from the growth of *P. taetrolens* was provided by differential temperature control. By lowering the culture temperature to 28 °C, higher cell densities were achieved while lactose oxidation was increased. The transition periods from the growth to production phase, accompanied by uncoupled overproduction of lactobionic acid, were shortened [6].

In order to increase LBA production, fermentation conditions and substrate availability for *P. taetrolens* growth should be optimised. Research shows how important the elemental content of the substrate is. Research work using acid whey, potassium, manganese and magnesium salts has shown that Mn^2+^ and Mg^2+^ ions are essential for the growth of *Pseudomonas taetrolens* [7].

An additional factor affecting lactobionic acid production is the dry matter content of the concentrated whey. Results from studies using acid whey show that a higher yield of LBA is obtained at a 20% concentration of total solids in the substrate. An increase in the total solids content of the substrate above 20% is not beneficial, slowing down the process due to the influence of minor whey compounds mostly in the form of minerals and their salts, while a higher concentration of lactose reduces the lactose dehydrogenase activity of *Pseudomonas taetrolens* [8].

The production of lactobionic acid by different types of microorganisms has been the subject of numerous studies. The ability to produce LBA has been demonstrated in bacteria of the genera *Acinetobacter* spp., *Psychrobacter* spp. and *Escherichia coli*, among others. Genetic engineering methods are being used to increase microbial productivity through heterologous expression of the quinoprotein glucose dehydrogenase from *Pseudomonas taetrolens*. The results confirm that expression of the lactose-oxidising enzyme in microorganisms is highly effective in enhancing their ability to produce lactobionic acid [1,9].

### 1.3. Characterisation of Whey in Production of Lactobionic Acid

Cheese whey is one of the most voluminous by-products of the dairy industry. Up to 50% of the whey generated is directly discharged into water systems, causing economic losses due to environmental pollution. Therefore, the food industry is trying to find a specific use for cheese whey in order to reduce environmental problems. Cheese whey can be subjected to various processes such as deproteinisation, microfiltration and ultrafiltration to produce concentrated whey proteins. The whey permeate obtained by these processes consists mainly of lactose (5%), water (93%) and minerals (0.53%), with minimal amounts of proteins (0.85%) and fats (0.36%). Such a poor composition limits its use; one way to use this by-product is to produce by microbial fermentation various compounds such as oligosaccharides, lactic acid and lactobionic acid (LBA) [10].

The production of acid whey has increased significantly in recent years due to the increased production of cottage cheese, yoghurt, Greek yoghurt and acid cheese. The high biological oxygen demand for acid whey and curd whey creates a problem in the management of this waste. The use of acid whey is also limited by its low pH, short shelf life and slightly salty taste. The composition of acid whey hinders the conversion of lactose to lactobionic acid by *Pseudomonas taetrolens*. By comparing the properties of sweet and sour whey, research is being conducted to increase the suitability of acid whey for LBA production. The amount of salt, protein concentration, pH, inoculum volume and culture time are important. The results showed that a higher concentration of protein and lactic acid in the medium negatively affects the oxidation of lactose to LBA, increasing the amount of acid whey in the medium can affect the yield of LBA, and a combination of sweet and acid whey can be a good solution for the biotechnological production of LBA using dairy waste. It is also important to note that repeated addition of *P. taetrolens* inoculum to the acid whey substrate optimises the conversion of lactose to LBA [2,11].

The suitability of acid whey for the production of lactobionic acid using a shake flask and bioreactor was tested and the results compared with sweet whey. It was shown that a bioreactor using sweet whey as substrate under controlled pH conditions is the most suitable method for the production of lactobionic acid using *Pseudomonas taetrolens*. In contrast, the use of acid whey as a substrate for lactose conversion is better in a bioreactor, and maintaining the fermentation medium pH at 6.5 determines the best fermentation conditions [12].

### 1.4. Flow Cytometry in Food Analysis

Flow cytometry is a test method that allows quantitative as well as qualitative analysis of cells in a short period of time. It is an improvement of the fluorescence microscope. Based on the analysis of laser light scanning a cell suspension, the physical and biological properties of cells can be quickly assessed. For this purpose, fluorescent dyes are used to examine various cellular parameters of microorganisms. In a flow cytometer, the prepared cell suspension enters the measurement zone, where it is exposed to a laser beam. The cells scatter the light, and the light simultaneously excites fluorochromes attached to the cell. The intensity of the light is measured using appropriate detectors [13].

The ability to assess morphological, phenotypic and functional characteristics has translated into the use of flow cytometry in many industries. It enables, among other things, phenotypic evaluation of peripheral blood, cerebrospinal fluid, bone marrow and lymph nodes or diagnosis of proliferative diseases of the circulatory system. It plays an important role in performing specialised immunological tests, assessing the immunophenotype and determining so-called multidrug resistance (chemotherapy).

Cytometric analysis is an extremely useful method in the microbiological analysis of foods. In probiotic products and starter cultures, enzymatic activity, the number of dead cells and the number of total cells of the genus *Lactobacillus* spp. were evaluated using appropriate dyes. Using cytometry, the effect of the freeze-drying process on the physiological state of *Lactococcus lactis* cells was also determined in order to select those strains that would perform better as starter cultures for cheese. However, most of the cytometric analysis is related to studies related to the detection of pathogenic microorganisms in food, including *Escherichia coli, Listeria* sp., *Salmonella* sp., *Legionella* sp. and *Staphylococcus aureus* in dairy products, eggs, water, poultry and beef. Receiving the results of the analysis in a short period of time allows quick action to be taken, which ultimately contributes to food safety in the food industry [14].

Studies were conducted using acid whey to produce lactobionic acid. Multiparametric flow cytometry was used to determine the physiological state of *P. taetrolens* to distinguish between live, dead and damaged cells during fermentation processes. Three trials were conducted with different amounts of inoculum (10% and 30% at the beginning of the process and 10% three times within 24 h). The trial with three times the amount of inoculum was distinguished by the highest yield and the best physiological state—the cytometry results showed that the bacteria were metabolically active and dominant throughout the fermentation process [2].

The aim of this research was to obtain the highest possible yield of lactobionic acid obtained in bioreactor cultures, to quantitatively analyse lactose and lactobionic acid by chromatographic methods and to investigate the prebiotic properties of lactobionic acid.

Obtaining the highest possible yield of lactobionic acid might provide an alternative to the production of this acid by biocatalytic production of lactobionic acid via enzymatic synthesis including enzyme purification. The novelty of this research lies in the use of new isolates of the *Pseudomonas taetrolens* strain and a microencapsulated form of these microorganisms. The aim is to obtain the highest possible yield of lactobionic acid from the waste product of the dairy industry—whey. In addition, research into the antimicrobial properties of lactobionic acid is being pioneered. There are also no reports in the literature on the preparation of synbiotic preparations with lactobionic acid and *Bifidobacterium*. Obtaining a high survival rate of these microorganisms in a human gastrointestinal tract model may contribute to new probiotic preparations with lactobionic acid (prebiotic).

The scope of this work includes the following:

- Culture and microencapsulation of bacteria of the *Pseudomonas taetrolens* species.
- Use of whey as a substrate in the microbiological production of lactobionic acid.
- Analysis of culture parameters: duration of culture, form of microorganisms (free and microencapsulated), concentration of lactose in the bioreactor (2% *w*/*v*) and effect of adding fresh, sterile whey on lactobionic acid production.
- Quantitative analysis of lactose and lactobionic acid using high-performance liquid chromatography.
- Analysis of metabolic activity of micro-organisms using flow cytometry with imaging.
- Culture of bacteria of the genus *Bifidobacterium*.
- Microencapsulation of Bifidobacteria with lactobionic acid and their placement in an in vitro model gastrointestinal tract.
- Survival analysis of microencapsulated bacteria and assessment of the prebiotic properties of lactobionic acid.

## 2. Results and Discussion

### 2.1. Antagonistic Activity of Lactobionic Acid

In our studies, we found the best LBA activity against two strains of *S. aureus* MSSA (methicillin-sensitive *Staphylococcus aureus*), one strain of MRSA (methicillin-resistant *Staphylococcus aureus*) and one strain of *C. albicans* (MIC 2 mg/mL). The activity of LBA against the second strains of MRSA and *C. albicans* was weaker, with an MIC of 5 mg/mL. For the remaining bacterial strains, the MIC was 5 mg/mL. For most yeast-like fungi, the effect was weaker and the MIC values were 5 or >5 mg/mL (Table 1).

Our observations presented that LBA exhibits weak antibacterial and antifungal activity.

### 2.2. Quantitative Analysis of Lactobionic Acid Obtained in Bioreactor Cultures

The first three cultures used free *Pseudomonas taetrolens* bacteria, a temperature set at 30 °C, pH = 6.25 and constant aeration (0.5 L/min).

The highest concentration of lactobionic acid was recorded at the 90th hour of culture and was 1.05 g/L, after the bacteria had previously stopped producing acid. The highest concentration of lactose was observed at the very beginning of the culture and was 5.62 g/L, then it began to gradually decrease until the 123rd hour. Acid production ended at 138 h (Table 2).

The highest concentration of lactobionic acid for culture 2 was 0.76 g/L; it was lower than for the first culture. Lactobionic acid began to be produced at the very beginning, but by the 42nd hour of culture, its concentration was already 0. Lactose concentration once again reached its highest value at the very beginning and was 8.57 g/L and gradually decreased (Table 3).

In the third culture (Table 4), a lactose solution was added at 71 h of culture, obtaining a 2% concentration of lactose in the bioreactor. The highest concentration of lactobionic acid was obtained at the 91st hour and was 1.36 g/L. It was higher than in the two earlier cultures, but the acid began to be produced later, after the addition of the lactose solution. The concentration of lactose was highest before the addition of the solution at the 22nd hour and was 21.80 g/L, falling until the 91st hour, where an increase in its concentration was observed, which translated into the amount of lactobionic acid obtained.

Three strains of bacteria from the genus *Pseudomonas taetrolens* were used to produce lactobionic acid in cultures 4–9:

1. *Pseudomonas taetrolens* 1
2. *Pseudomonas taetrolens* 4
3. *Psedomonas taetrolens* 4′

Both free and microencapsulated microorganisms were used, and the effect of 2% lactose concentration in the bioreactor and the addition of fresh sterile whey on lactobionic acid production was studied. Other parameters such as pH, temperature and aeration remained unchanged.

In the fourth culture (Table 5), *Pseudomonas taetrolens* strain 1 was used in microcapsule form. At the 166th hour of culture, a solution of fresh whey was added. The highest concentration of lactobionic acid was reached at the 54th hour of culture and was 2.74 g/L. After that, the concentration of the acid began to decrease, only to increase again after 190 h of culture. The situation is similar for lactose, after a decrease in concentration, its amount began to increase after the addition of fresh whey.

The fifth culture used free bacteria from *Pseudomonas taetrolens* strain 4 (Table 6). Fresh whey solution was added at 193 h of culture. The highest concentration of lactobionic acid was recorded at 247 h and was 0.80 g/L. As in the earlier culture, the addition of fresh whey solution was followed by an increase in lactose concentration, which further translated into an increase in the amount of lactobionic acid concentration in the bioreactor.

In culture No. 6, microencapsulated bacteria from *Pseudomonas taetrolens* strain 4 were used and fresh whey solution was added at 231 h of culture. The highest amount of lactobionic acid was observed at the 44th hour of culture and was 1.50 g/L. Despite the addition of whey solution. the lactose content in the bioreactor did not change. The production of acid ended at 275 h; the concentration of lactose gradually decreased (Table 7).

The seventh culture used free bacteria from *Pseudomonas taetrolens* strain 1 and no fresh whey solution was added (Table 8). The highest concentration of lactobionic acid was recorded at the 24th hour of culture and was 1.72 g/L. Despite the high concentrations of lactose. The amount of lactobionic acid produced was not high. The bacteria finished producing acid at the 113th hour of culture.

In the eighth and ninth cultures for the *Pseudomonas taetrolens* 4′ strain, the concentration of lactose in the bioreactor was 2% from the very beginning. In both cultures, fresh whey solution was not added.

In culture No. 8, free bacteria of *Pseudomonas taetrolens* 4′ strain were used, the concentration of lactobionic acid gradually increased, and reached its highest value in the last 102 h of culture: 37.42 g/L (Table 9). The concentration of lactose decreased proportionally to the increase in the concentration of lactobionic acid in the bioreactor. For culture No. 8, where the lactose concentration in the bioreactor was 2% from the start, the highest lactobionic acid concentration result was recorded, so the amount of lactose in the whey has an impact on acid production. It is likely that such high production of lactobionic acid is influenced by the type of *P. taetrolens* strain used. This is a pure isolate from a single cell of *P. taetrolens* DSM 21104. Perhaps the enzyme activity of this strain is markedly higher than that of the other strains.

In the ninth culture, microencapsulated bacteria of the *Pseudomonas taetrolens* 4′ strain were used (Table 10). The concentration of lactobionic acid reached a maximum at the 141st hour of culture and was 1.98 g/L. The concentration of lactose in the bioreactor was highest at the beginning and its value was 44.68 g/L and with time it began to decrease. In culture 9, after using microencapsulated *Pseudomonas taetrolens* 4′ bacteria, it was observed that the yield of lactobionic acid decreased significantly compared to the use of free *Pseudomonas taetrolens* 4′ bacteria. The reason for this phenomenon may be the insufficient permeability of the capsule walls to the enzymes produced by *P. taetrolens*.

**Figure 1:**
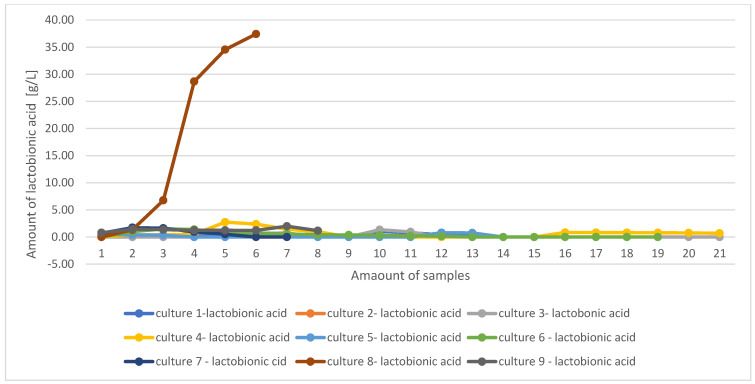
Comparison of changes in lactobionic acid content for 9 cultures.

In each culture, a decrease in lactose concentration was observed over time, indicating that the microorganisms were metabolising this substrate. After the addition of fresh sterile whey, an increase then a decrease in lactose concentration was observed in the bioreactor. A decrease in lactose concentration correlates with an increase in lactobionic acid concentration. This is ideally represented in Table 9, which shows lactobionic acid and lactose concentrations in culture No. 8. The research conducted also proved the effect of lactose concentration in the bioreactor on lactobionic acid synthesis. For culture No. 8, where the added lactose concentration in the bioreactor was 2% from the start, the highest lactobionic acid concentration result was recorded, so the amount of lactose in the whey has an impact on acid production. A comparison was made between the two cultures in bioreactors with a 2% added lactose concentration run with the same *Pseudomonas taetrolens* 4′ strain, but in two forms: free and microencapsulated, in order to assess the effect of alginate capsules on lactobionic acid production. The results obtained, however, showed that microencapsulation of bacteria does not favour lactobionic acid synthesis. The same conclusions can be drawn for the rest of the cultures. In none of them was microencapsulation shown to promote acid production by the microorganisms (Figure 1).

After statistical analysis comparing the amounts of lactobionic acid produced in the studied cultures using the Kruskal–Wallis test (due to the lack of normal distribution of the studied variables) at the significance level α = 0.05, statistically significant differences in the amount of lactobionic acid in the studied cultures are found. Culture No. 8 had the highest mean in acid content (18.12 g/L) and the highest standard deviation value (17.28), indicating that the amount of lactobionic acid increased significantly during culture.

By running nine bioreactor cultures, the best environmental conditions were selected: pH = 6.25, temperature 30 °C, continuous aeration (0.5 L air/min) and a lactose added concentration in the bioreactor of 2%. The best strain for microbial synthesis of LBA proved to be *Pseudomonas taetrolens* 4′ in the unencapsulated free form. Such selected culture parameters led to the highest concentration of lactobionic acid (37.42 g/L).

The aim of the previous research was to develop a method of lactobionic acid production from whey using bacteria of the species *Pseudomonas taetrolens* DSM 21104 [15]. Analyses of the lactobionic acid production method from whey showed that the following factors have a significant effect on its efficiency: the frequency of whey batch feeding, pH and the type of bacteria application, i.e., microencapsulated vs. free. The highest concentration of lactobionic acid of 22.03 mg/mL was obtained when whey was batch fed at 72 h intervals, pH was maintained at 6.25 and bacteria were enclosed in alginate microcapsules [15]. Comparing the results obtained in the present experiments with those of previous experiments, the use of a pure *P. taetrolens* strain yielded 69.86% more lactobionic acid when cultured on whey.

### 2.3. Analysis of Metabolic Activity of Microorganisms Using Flow Cytometer with Imaging

**Figure 2:**
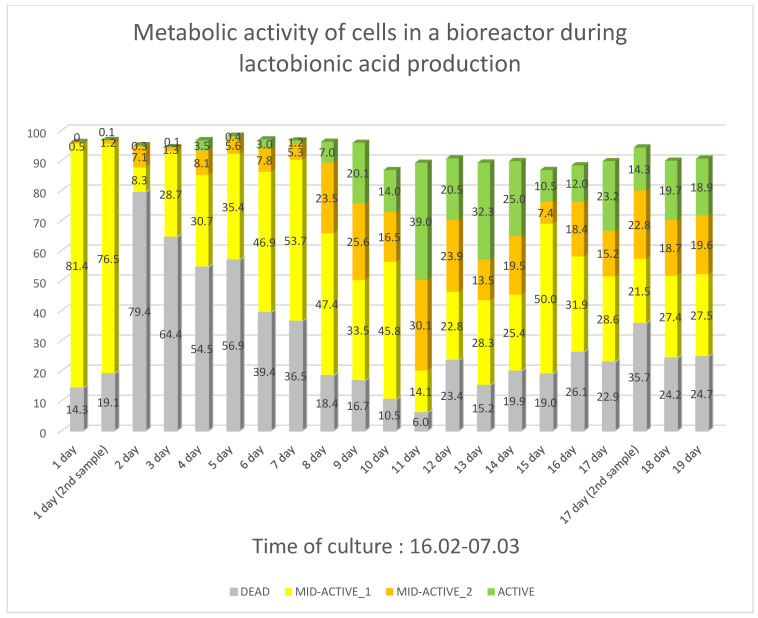
Comparison of metabolic activity of microorganisms in a bioreactor.

Analysis of the metabolic activity of microorganisms in the bioreactor using a flow cytometer with imaging was carried out for culture 5 for *Pseudomonas taetrolens* strain 4 in free (non-encapsulated) form (Figure 2).

The percentage of bacteria exhibiting low metabolic activity (high intensity of fluorescence signals from the RedoxSensor Green reagent) with concomitant reduced cell membrane integrity (high fluorescence intensities from P) was calculated. The highest number of these microorganisms was recorded on day 2 of culture, after which it began to decrease.

Cells characterised by reduced metabolic activity (low intensity fluorescence signals from RedoxSensor Green reagent) with concomitant cell membrane integrity (low fluorescence intensities from PI) were marked in yellow (MID-ACTIVE_1). The highest number of these cells was observed on day 1 of culture. This number also started to decrease over time.

Cells characterised by metabolic activity (high fluorescence signals from the RedoxSensor Green reagent) with reduced cell membrane integrity (high fluorescence intensities from PI) were marked in orange (MID-ACTIVE_2). The maximum of these microorganisms was recorded on day 11 of culture.

In contrast, cells distinguished by metabolic activity (high fluorescence signals from RedoxSensor Green reagent), with concomitant cell membrane integrity (low fluorescence intensities from PI), were marked in green (ACTIVE). The highest number of these bacteria was recorded on day 11 of culture and they were present until the end of the culture in the bioreactor.

By observing the physiological state of the microorganisms in the bioreactor, a change in their metabolic activity is apparent (Table 11). At the very beginning of the culture, most bacteria are characterised by low metabolic activity, lactobionic acid being produced despite the high concentration of lactose only up to day 3 of the culture. It is likely that the increase in the concentration of dead cells after day 2 of culture is due to the microorganisms adapting to the new environmental conditions—the new medium, the amount of oxygen present in the whey and the acidity of the environment. Adaptation of the bacteria is followed by a gradual decrease in the number of cells with low metabolic activity. After the addition of whey (day 9 of culture), an increase in the number of cells showing metabolic activity is observed. On day 11 of the culture, when the highest number of cells with metabolic activity with cell membrane integrity (ACTIVE) is present in the bioreactor, the highest concentration of lactobionic acid is also observed for this culture. It is also significant that on day 11 of culture, the lactose concentration was much lower than at the start of culture. The results obtained show how important (in addition to the selected environmental conditions) the presence of metabolically active live microorganisms is for the production of lactobionic acid.

### 2.4. Survival of Bifidobacterium Bifidum DSM 20239, DSM 20082, DSM 20215 and DSM 20456 Microencapsulated with Lactobionic Acid in In Vitro Model of Gastrointestinal Tract

**Figure 3:**
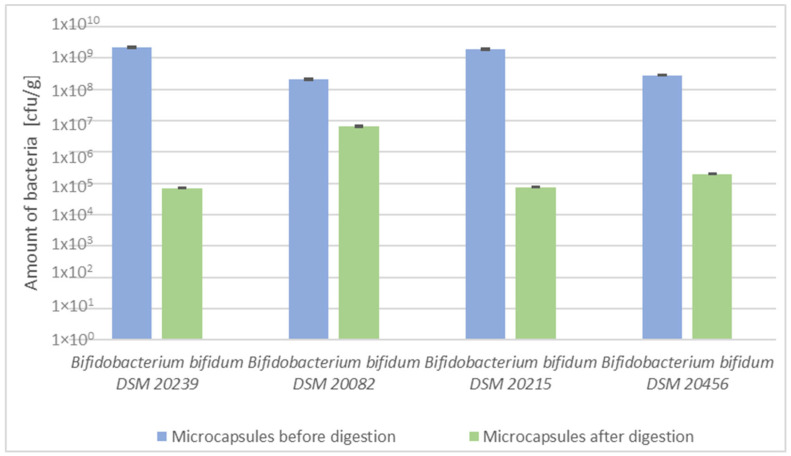
The number of viable cells of microorganisms encapsulated in microcapsules before and after the digestion process.

Figure 3 shows a comparison of the number of probiotic microorganisms of the genus *Bifidobacterium* before and after the in vitro digestion process. The highest number of microorganisms in the microcapsules before the digestion process was obtained by *Bifidobacterium bifidum* DSM 20239 (2.2 × 10^9^ cfu/g), while the lowest was obtained by *Bifidobacterium bifidum* DSM 20082 (2.08 × 10^8^ cfu/g). After exposure to low pH at the stomach stage, the lowest number of released microorganisms was distinguished by *Bifidobacterium bifidum* DSM 20456 with 4.25 × 10 cfu/mL, followed *by Bifidobacterium bifidum* DSM 20215 (2.5 × 10^2^ cfu/mL) and *Bifidobacterium bifidum* DSM 20082 (4.0 × 10^2^ cfu/mL), and the highest number by *Bifidobacterium bifidum* DSM 20239 strain: 1.0 × 10^3^ cfu/mL. After the intestinal stage with a pH of 7.4, *Bifidobacterium bifidum* strain DSM 20456 once again had the lowest number of viable cells (3.75 × 10 cfu/mL), followed by *Bifidobacterium bifidum* DSM 20215 (3.0 × 10^2^ cfu/mL). In contrast, *Bifidobacterium bifidum* DSM 20082 (1.9 × 10^6^ cfu/mL) stood out as having the highest number of microorganisms. The highest number of viable cells in the microcapsules after the digestion process was obtained by the *Bifidobacterium bifidum* DSM 20082 strain with a result of 6.5 × 10^6^ cfu/g, and the lowest by *Bifidobacterium bifidum* DSM 20239 (7.0 × 10^4^ cfu/g) (Table 12).

The evaluation of the survival of probiotic bacteria of the genus *Bifidobacterium* encapsulated in microcapsules with lactobionic acid acting as a prebiotic led to satisfactory results. The created microcapsules took the form of a synbiotic, where the prebiotic improves the survival and colonisation of probiotics in the gastrointestinal tract. Although *Bifidobacterium bifidum* strain DSM 20082 achieved the lowest number of viable cells in the microcapsules before digestion, it achieved the highest number of viable microorganisms in the microcapsules after in vitro digestion. The bacteria of this strain began to be released already at the stomach stage, to be released to an even greater extent after the intestinal stage at pH 7.4. Such conditions in the in vitro model digestive tract are similar to those in the large intestine—the target site of probiotic action. The survival of probiotic bacteria in the gut is extremely important because of the functions they perform. They inhibit the growth of pathogenic microflora and are responsible for human health and physiology. The importance of the microbiome in the functioning of the brain and central nervous system, emotions and cognitive function is increasingly being discussed. After statistical analysis comparing the number of viable cells in the microcapsules before digestion and in the microcapsules after digestion using the Wilcoxon test (due to the lack of normal distribution of the variables tested) at the significance level α = 0.05, it was found that there are significantly statistical differences between the number of microcapsules before and after in vitro digestion (*p* = 0.05). However, the release of bacterial cells on the outside of the capsules during the digestion process was observed and the total number of microorganisms after the whole process was not drastically reduced.

In previous studies, the prebiotic properties of lactobionic acid in the human gastrointestinal model were tested. Five different strains of probiotic, or potentially probiotic, bacteria were used in the microencapsulation process; these were *Lactobacillus casei* Shirota, *Lactococcus lactis* ATCC1, *Lactobacillus fermentum*, *Bifidobacterium bifidum* DSM 20456 and *Bifidobacterium bifidum* DSM 20082. The alginian microcapsules with starch (4% (*w*/*v*) and a degree of substitution of 0.03) functioned as a protective barrier for the probiotic microorganisms closed in them, protecting them from adverse conditions in the human digestive tract. The capsule solution began when a pH of 7.4 was reached; this corresponded to pH in the target probiotic site, an in vitro model of the colon. The capsules had completely dissolved after 24 h of digestion at a pH of 8. After microencapsulation, the microorganisms could survive the in vitro model digestion process while retaining the ability to produce biomass. Factors such as pH and time affect the solution of alginate microcapsules. The addition of lactobionic acid stimulated the growth of probiotic and potentially probiotic bacteria, thus confirming its prebiotic properties [16].

The effect of microencapsulation by alginate and alginate–chitosan on the survival of *Saccharomyces boulardii* ATCC MYA796 in simulated gastric and intestinal juices was investigated by Niamah et al. [17]. The percentage survival of encapsulated *S. boulardii* was 80% and 85% after 240 min for the alginate microcapsules method and alginate–chitosan microcapsules method while the percentage survival of *S. boulardii* free cells was 67%. The microencapsulation of *S. boulardii* cells does prevent the effect of acid and bile salts [17]. Our study focused on anaerobic microorganisms and therefore their survival rate in the gastrointestinal tract model is lower than that of aerobic microorganisms. In this study, the access of oxygen is limited but cannot be completely eliminated.

The results obtained also prove the protective properties of alginate microcapsules, which protected the cells of *Bifidobacterium* from the harsh conditions of the digestive tract and allowed them to be released at the given stages of digestion, lactobionic acid showed prebiotic properties. Further in vitro studies using LBA as a prebiotic substance, given its poor absorption capacity at the small intestinal stage and resistance to digestive enzymes, may help increase its popularity and prove its functional properties.

## 3. Materials and Methods

### 3.1. Microorganisms

Four different strains of probiotic or potentially probiotic bacteria were used for the microencapsulation process.

1. *Bifidobacterium bifidum* DSM 20239 (DSMZ-German Collection of Microorganisms and Cell Cultures GmbH, Braunschweig, Germany)

2. *Bifidobacterium bifidum* DSM 20082 (DSMZ-German Collection of Microorganisms and Cell Cultures GmbH, Germany)

3. *Bifidobacterium bifidum* DSM 20215 (DSMZ-German Collection of Microorganisms and Cell Cultures GmbH, Germany)

4. *Bifidobacterium bifidum* DSM 20456 (DSMZ-German Collection of Microorganisms and Cell Cultures GmbH, Germany)

5. *Pseudomonas taetrolens* DSM 21104 (DSMZ-German Collection of Microorganisms and Cell Cultures GmbH, Germany)

MRS Broth medium (Oxoid, Altrincham, England) was used to multiply the above probiotic microorganisms.

### 3.2. Substances Used for Microencapsulation of Bifidobacterium

The following substances were used in the microencapsulation process:

- Cationic starch (Central Potato Industry Laboratory, Luboń, Poland) with a degree of substitution of 0.03 and a concentration of 8%
- Calcium chloride with a concentration of 1.22% (POCH, Gliwice, Poland)
- Alginic acid sodium salt with a concentration of 0.6% (Sigma-Aldrich, St. Louis, MO, USA)
- Bidistilled water
- Lactobionic acid (Sigma-Aldrich, St. Louis, MO, USA) (Table 13)

Sterile sodium citrate 3% (*w*/*v*) was used to dissolve the finished microcapsules (POCH, Gliwice).

### 3.3. Substances Used During In Vitro Digestion

The substances used during digestion in the gastrointestinal model were the following:

- Pepsin: 0.0453 g was dissolved in 1 mL of 0.1M HCl (Sigma-Aldrich).
- Pancreatin: 0.01 g and bile acid salts 0.06 g were dissolved in 5 mL of 0.1M NaHCO_3_ (Sigma-Aldrich).
- 100 mL MRS Broth (Oxoid, England).

### 3.4. Methods

#### 3.4.1. Preparation of Inoculum for Microencapsulation of *Bifidobacterium*

The inoculum of probiotic bacteria consisted of microorganisms from a 100 mL stationary culture in MRS Broth medium (Oxoid, England). The inoculum was propagated for 48 h at 37 °C and under anaerobic conditions. The resulting biomass was centrifuged for 15 min at 4000 rpm (MPW 350R, MPW Med. Instruments, Warszawa, Poland).

#### 3.4.2. Microencapsulation of Probiotic Bacteria

Microencapsulation was carried out in a microbiology laboratory under sterile conditions using the method described by Goderska and Czarnecki in 2008 [18].

The preparation of solutions was as follows:

(a) An amount of 0.61 g of CaCl_2_ was weighed, then the volumetric flask was made up to 100 mL with distilled water. After dissolving the calcium chloride, 8 g of cationic starch was poured in.
(b) A total of 1.22 g CaCl_2_ was weighed into a volumetric flask and the flask was then made up to 100 mL with distilled water.
(c) Finally, 0.6 g of sodium alginate was weighed into a volumetric flask and the flask was made up to 100 mL with distilled water. Due to the difficult dissolution of alginate, the prepared solution was slightly heated.

All solutions were prepared on a magnetic stirrer with a heating option. The finished solutions were sterilised at 121 °C for 15 min.

To the centrifuged biomass, 20 mL of 8% cationic starch was added. Once the biomass was thoroughly dissolved in the starch, 2.2 mL of sterile lactobionic acid was added at the appropriate concentration for the strain under study. The prepared biomass was collected into a sterile syringe, onto which a 0.8 mm diameter needle was inserted, the kit thus prepared was placed at a height of approximately 5 cm, and the contents were dropped into the stirring sodium alginate solution.

The resulting capsules were drained using a pre-sterilised strainer and washed profusely with sterile bidistilled water. The microcapsules prepared in this way were transferred to the calcium chloride solution to harden for 15 min with constant stirring. After this time, the cured capsules were again drained and washed with sterile bidistilled water.

#### 3.4.3. Determination of Number of Live Cells Encapsulated in Microcapsules

A collected sample of 1 g of microcapsules containing *Bifidobacterium* was placed in a sterile 3% (*w*/*v*) sodium citrate solution of 100 mL for 15 min to dissolve the capsules. While the microcapsules were dissolving, a series of decimal dilutions from 10^−1^ to 10^−8^ were prepared. When determining the number of viable cells, the flooding method was used. An amount of 1 mL of the prepared dilutions was applied to the previously prepared Petri dishes and then flooded with liquid MRS Agar (BTL, Łódź, Poland). After solidification, the plates were incubated for 48 h under anaerobic conditions at 37 °C. After the incubation time, the culture results were read by counting bacterial colonies.

#### 3.4.4. Analysis of Obtained Microencapsulated Synbiotic Preparations with Lactobionic Acid

The survival analysis of *Bifidobacterium* encapsulated in microcapsules with lactobionic acid was conducted in an in vitro model gastrointestinal tract including the stomach and small intestine. Digestion of the synbiotic preparations was carried out in a 1 L glass vessel. The whole vessel was closed with a glass cover having holes for the pH electrode, thermometer and glass tube, which was used for sampling during the digestion process. The cover had a sliver on which was placed a cap with capillaries through which reagents were dispensed. The entire set-up was placed in a 37 °C water bath and on a magnetic stirrer [16].

Under sterile conditions, 14 g of microcapsules containing one of the tested strains of *Bifidobacterium* was weighed into a glass vessel and poured into 100 mL of sterile MRS Broth medium (BTL, Łódź, Poland). The vessel was placed in a water bath set at 37 °C.

##### Stomach Stage

The pH of the mixture was lowered to pH = 2 with 1M HCl by dropping in a calculated dose of acid using a peristaltic pump. Once the adjusted pH was achieved, 1 mL of previously prepared pepsin was added to the mixture. The digestion was carried out for 2.5 h on a magnetic stirrer and at 37 °C. After the allotted time, a sample of the liquid medium was taken from which a series of decimal dilutions were made from 10^−1^ to 10^−3^.

##### Small Intestine Stage

When the pH reached 6 within 30 min, a previously prepared pancreatic–intestinal extract of 5 mL was added. 1M NaHCO_3_ was dosed into the mixture using a pump until a pH of 7.4 was reached. As with the stomach stage, the digestion process was carried out at 37 °C on a magnetic stirrer for a total time of 2.5 h. After this time and a pH of 7.4. 10 mL of sample and microcapsules that had not dissolved were collected.

#### 3.4.5. Determination of Number of Viable Cells During In Vitro Digestion

After the allotted time had elapsed and pH = 7.4 had been reached, a sample of the liquid medium was taken from which a series of decimal dilutions were made from 10^−1^ to 10^−3^; a sample of microcapsules was also taken and dissolved in a 3% sodium citrate solution. After 15 min had elapsed and the microcapsules had dissolved, a series of decimal dilutions from 10^−1^ to 10^−5^ were made.

The dilutions prepared in this way (from 10^−1^ to 10^−5^) were applied in 1 mL to Petri dishes and flooded with liquid MRS Agar (BTL, Łódź, Poland). After solidification, the plates were incubated under anaerobic conditions for 48 h at 37 °C. After a given time, the cultures were read by counting the number of microbial colonies.

#### 3.4.6. Preparation of Inoculum of *P. taetrolens* DSM 21104 (Free and Microencapsulated) for Production of Lactobionic Acid in Bioreactor

Three strains of bacteria of the genus *Pseudomonas taetrolens* were used for the production of lactobionic acid:

1. *Pseudomonas taetrolens* 1
2. *Pseudomonas taetrolens* 4
3. *Pseudomonas taetrolens* 4′

Microorganisms were grown in a 100 mL stationary culture using Trypticase Soy Broth (Oxoid, England). The process was carried out in a shaker at a stirring speed of 200 rpm for 48 h at 30 °C. The resulting biomass was centrifuged for 15 min at 10,000 rpm (MPW 350R, MPW Med. Instruments, Warszawa, Poland). The separated biomass was then added to sterile saline solution of 100 mL (for free bacteria) or to sterile 4% cationic starch solution containing 100 mM CaCl_2_ (microencapsulated bacteria).

The microencapsulation of bacteria of the genus *Pseudomonas* spp. involved using the following reagents:

- Cationic starch 4% *w*/*v* (Central Laboratory of the Potato Industry, Luboń, Poland).
- Alginic acid sodium salt (Sigma-Aldrich, St. Louis, MO, USA).
- Calcium chloride (POCH, Gliwice, Poland).
- Sodium citrate 3% *w*/*v* (POCH, Gliwice, Poland).

Microencapsulation was carried out in a microbiology laboratory under sterile conditions using the method described by Goderska and Czarnecki in 2008 [18].

For the microencapsulation process, an inoculum of *P. taetrolens* bacteria derived from a 100 mL stationary culture in Trypticase Soy Broth (Oxoid, England) was used. The culture was grown for 48 h at 37 °C in a shaker at a stirring speed of 200 rpm. The resulting biomass was centrifuged for 15 min at 4000 rpm (MPW 350R, MPW Med. Instruments, Warszawa, Poland). A sterile 4% *w*/*v* cationic starch solution containing 100 mM CaCl_2_ was added to the obtained biomass. The resulting solution was then placed in a sterile syringe and dropped into a 0.6% *w*/*v* sodium alginate solution. The whole process took 15 min on a magnetic stirrer at 500 rpm. After the given time, the microcapsules were carefully washed with bidistilled water and placed in calcium chloride solution for 15 min. The cured microcapsules were drained on a sieve and washed again with bidistilled water. The amount of *Pseudomonas taetrolens* in microcapsules was 8 × 10^8^ cfu/g.

#### 3.4.7. Preparation of Substrate for Lactobionic Acid Biosynthesis

For the production of lactobionic acid by microbial oxidation of lactose, powdered rennet whey (OSM Company TOP TOMYŚL, Nowy Tomyśl, Poland) was used. In total, 27.8 g of powdered whey was weighed and dissolved in 1000 mL of distilled water (2% lactose). The prepared solution was sterilised at 121 °C for 15 min.

#### 3.4.8. Preparation of Lactobionic Acid in Bioreactor

For the microbiological conversion of lactose to lactobionic acid, a 2 L glass bioreactor equipped with a pH electrode, stirrer, thermometer, heating jacket, oxygen electrode and air inlet filter was used to ensure aerobic conditions. In addition, the vessel was equipped with pumps to provide dosing of the nutrient solution, base (1M NaOH) and acid (1M HCl), which regulated the pH. A total of 1000 mL of sterilised whey (medium) was placed in the bioreactor and inoculated with a 100 mL suspension of *P. taetrolens* bacteria in saline (2.3 × 10^8^ cfu/mL). Lactobionic acid biosynthesis was carried out at 30 °C with a stirring speed of 120 rpm, pH maintained at 6.25 and continuous aeration (0.5 L air/min).

Variants of culture:

Culture No. 1—free *Pseudomonas taetrolens* DSM 21104; culture time: 138 h

Culture No. 2—free *Pseudomonas taetrolens* DSM 21104; culture time: 116 h

Culture No. 3—free *Pseudomonas taetrolens* DSM 21104; culture time: 114 h; lactose was added at 71 h of culture, obtaining a 2% concentration in the bioreactor

Culture No. 4—microencapsulated *Pseudomonas taetrolens* 1; culture time: 334 h; 315 mL of culture was collected and 500 mL of fresh whey was added at 166 h

Culture No. 5—free *Pseudomonas taetrolens* 4; culture time: 284 h; 325 mL of culture was collected and 500 mL of fresh whey was added at 193 h

Culture No. 6—microencapsulated *Pseudomonas taetrolens* 4; culture time: 421 h; 315 mL of culture was collected and 500 mL of fresh whey was added at 231 h

Culture No. 7—free *Pseudomonas taetrolens* 1; culture time: 138 h

Culture No. 8—free *Pseudomonas taetrolens* 4′; culture time: 102 h; the concentration of added lactose in the bioreactor was 2% from the very beginning

Culture No. 9—microencapsulated *Pseudomonas taetrolens* 4′; culture time: 166 h; the concentration of added lactose in the bioreactor was 2% from the very beginning

A total of nine bioreactor cultures were carried out. It was checked which parameters influence acid production and to what extent. Variable culture parameters included the duration of culture, the form of microorganisms (free and microencapsulated), the concentration of lactose in the bioreactor (2% *w*/*v*) and the effect of adding fresh, sterile whey. Samples for the study were taken daily into Eppendorfs and then frozen (5 × 2 mL = 10 mL/day).

#### 3.4.9. Determination of Lactose and Lactobionic Acid

##### Preparation of Samples for Chromatographic Analysis

Frozen samples were thawed and then centrifuged in a centrifuge for 15 min at 10,000 rpm at 4 °C (MPW 350R. MPW Med. Instruments, Poland). Depending on the addition of the lactose solution, 10-fold or 20-fold dilutions of the culture fluid were prepared. Samples were filtered through a 0.45 µm syringe filter.

##### Quantitative Analysis of Lactose and Lactobionic Acid Using High-Performance Liquid Chromatography

Lactose and lactobionic acid were measured by HPLC using the Rezex ROA Organic Acid column (300 × 7.8 mm; Phenomenex International, Torrance, CA, USA) at 55 °C with detection at 210 nm; the RI detector and PAD detector had an eluent of 0.025 M sulfuric acid at 0.5 mL/min. The amount of lactose and acid was determined using the ratio resulting from the changing amount of liquid medium (whey) in the bioreactor. Results are presented in g/L [15].

#### 3.4.10. Analysis of Microorganisms Using Flow Cytometer with Imaging

Fluorescent dye array:

Cytometric analysis of microbial cell viability and metabolic activity, expressed as oxido-reduction potential, was performed using the Redox Sensor™ Green Vitality Kit (Thermo Scientific, Osterode Am Harz, Germany) containing the following reagent:

- BacLight™ Redox Sensor™ Green
- Fluorochrome PI (propidium iodide)

The purpose of the PI fluorescent dye was to assess cell viability based on cell membrane integrity (fluorescence in the red band − emission maximum = 636 nm).

The Redox Sensor™ Green reagent functioned as an indicator of cellular oxidoreductive potential, providing a substrate for cellular enzymes of the oxido-reductase group. The median signal intensities of the green fluorescence (fluorescence in the green band − emission maximum = 520 nm) emitted by the enzyme conversion products of the Redox Sensor™ Green reagent are directly proportional to the redox potential of the analysed cells. This makes it possible to determine the metabolic activity of microbial cells.

Preparation of samples for cytometric analysis:

Sample preparation for analysis included pre-centrifugation (1100× *g*, 30 s) of 500 µL of each sample. A total of 100 µL of the supernatant was collected and centrifuged (3000× *g*, 5 min). The supernatant this time was discarded and the pellet was resuspended in 1 mL PBS solution (1%). A 250 µL suspension of microorganisms was used for staining. Further, 1.5 µL of Redox Sensor™ Green reagent and 0.5 µL of propidium iodide (PI) were added to each sample. Samples were incubated without light for 10 min.

Assessment of cell metabolic activity using flow cytometry:

Analysis of the prepared samples was performed using an Amnis^®^FlowSight^®^ imaging flow cytometer (Luminex Corp., Austin, TX, USA) (Department of Microbiology and Food Biotechnology, Poznań University of Life Sciences) equipped with the following:

- 3 lasers (405 nm, 488 nm and 642 nm).
- 5 fluorescence channels (multi-channel CCD camera acquisition)
- a laser-scattered–side-scatter (SSC) detector.

A 488 nm blue laser was used for the excitation of both fluorescent reagents used in the analysis.

A three-stage morphological characterisation of the microbial cells analysed was carried out:

(1) In the first step, Gradient RMS and Contrast parameters were used to define images of cells located in a well-defined plane of focus with high resolution and contrast.
(2) In the second stage, the intensity of the Ch2 channel green fluorescence (RSG) signals from the Redox Sensor™ Green reagent was used in the form of the Intensity parameter (discrimination of cells from a non-cellular background—a gate defining cells covering low median values of fluorescence intensity from Redox Sensor™ Green).
(3) The third stage used parameters: Aspect Ratio and Area, derived from the digital image processing of cells in the bright field of view, and related to the shape and size of the analysed cells, respectively.

Cytometric analysis was performed based on specific laser settings and conversion of the collected fluorescence signals to a logarithmic scale. Data were analysed using IDEAS®version 6.3.23.0 software (Luminex Corp., Austin, TX, USA). The Intensity parameter was used to assess the intensity of the green fluorescence (channel 2) derived from the RedoxSensor Green reagent, and the intensity of the red fluorescence (channel 5) derived from the PI fluorochrome. Cell sub-populations were then defined by gating on a dot plot of the relationship between green fluorescence (channel 2) and red fluorescence (channel 5).

The analysis allowed the detection of non-active (dead), those showing intermediate levels of metabolic activity and active sub-populations of microbial cells. Determination of the oxido-reduction potential of the microbial cells was based on the median values of the green fluorescence signals (channel 2) of the 3 sub-populations (active. intermediate and dead) defined by a two-dimensional plot of the relationship between green fluorescence intensity (channel 2) and red fluorescence intensity (channel 5). Each sample was analysed in triplicate [19].

#### 3.4.11. Analysis of Antagonistic Activity of Lactobionic Acid

The tests were performed on 6 bacterial (*Staphylococcus aureus*, *S. aureus* methicillin-resistant (MRSA). *S. epidermidis*, *Escherichia coli*, *Pseudomonas aeruginosa* and *Proteus mirabilis*) and 6 fungal yeast-like species (*Candida albicans*, *C. tropicalis*, *C. parapsilosis*, *C. glabrata*, *C. krusei*= and *Rhodotorula rubra*). For each species, 2 strains were tested. Bacteria were grown in tryptone soy agar (Graso Biotech, Starogard Gdański, Poland) and fungi in Sabouraud dextrose agar (Graso Biotech, Starogard Gdański, Poland). The antimicrobial activity was studied as minimal inhibitory concentrations (MICs). MICs of LBA were determined by the micro-dilution method using 96-well plates (Nest Scientific Biotechnology, Wuxi, China), as previously shown [20]. In the studies, we used inocula in a final concentration of 10^6^ CFU/mL. Serial dilutions of LBA were performed to obtain concentrations ranging from 5000 to 63 µg/mL. The plates were incubated at 35 °C for 24 h. The MIC value was the lowest LBA concentration that inhibited any microbial growth.

#### 3.4.12. Statistical Analysis

The number of bacteria was counted in triplicate using the plate method. Excel 2000 was used to statistically analyse all the experimental designs using mean descriptive statistics and single-factorial analysis of variance for *p* < 0.05.

## 4. Conclusions

Probiotic microorganisms of the genus *Bifidobacterium* spp. survived the microencapsulation process and the in vitro digestion process. Alginate microcapsules provided a sufficient protective barrier for the encapsulated probiotic bacteria together with lactobionic acid, taking the form of a synbiotic. The release of bacteria from the capsules at different stages of digestion demonstrates the permeability of the microcapsules and the possibility of colonisation of the large intestine by probiotics. The addition of lactobionic acid stimulated the growth of probiotic bacteria, proving its prebiotic properties. By conducting nine bioreactor cultures, the highest lactobionic acid yield was obtained for culture 8 and was 37.42 g/L, selecting the appropriate strain and environmental conditions. The highest lactobionic acid yield was demonstrated for 2% lactose concentration in the bioreactor. The addition of fresh, sterile whey stimulated lactobionic acid production. Whey was a valuable source of lactose for *Pseudomonas taetrolens* bacteria. Of the three *Pseudomonas taetrolens* strains tested, 1, 4 and 4′, only the last strain performed well in obtaining high concentrations of lactobionic acid. Microencapsulated *Pseudomonas taetrolens* bacteria did not stimulate lactobionic acid production in sufficient quantity. The presence of metabolically active cells with cell membrane integrity is necessary for microbial production of lactobionic acid.

## Figures and Tables

**Table 1:** MIC values of lactobionic acid (LBA) against tested strains.

Microbial Species (Two Strains from Each)	LBA MIC (mg/mL)
*Staphylococcus aureus*	2
*S. aureus methicillin-resistant* (MRSA)	2–5
*S. epidermidis*	5
*Escherichia coli*	5
*Pseudomonas aeruginosa*	5
*Proteus mirabilis*	5
*Candida albicans*	2–5
*C. tropicalis*	>5
*C. parapsilosis*	5
*C. glabrata*	>5
*C. krusei*	>5
*Rhodotorula rubra*	5

**Table 2:** Concentration of lactobionic acid and lactose for culture No. 1.

Time [h]	Lactose [g/L] ± SD	Lactobionic Acid ± SD [g/L]
0	5.62 ± 0.01	0.00 ± 0
15	5.54 ± 0.01	0.00 ± 0
21	5.43 ± 0.01	0.00 ± 0
26	4.96 ± 0.01	0.54 ± 0.001
41	4.76 ± 0.01	0.61 ± 0.002
45	4.35 ± 0.01	0.46 ± 0.001
49	3.44 ± 0.007	0.00 ± 0
65	1.64 ± 0.003	0.00 ± 0
74	1.39 ± 0.003	0.00 ± 0
90	0.63 ± 0.002	1.05 ± 0.003
98	0.36 ± 0.001	0.78 ± 0.002
115	0.27 ± 0.001	0.42 ± 0.001
123	0.00 ± 0	0.12 ± 0.001
138	0.00 ± 0	0.00 ± 0

Free *Pseudomonas taetrolens* DSM 21104; culture time: 138 h.

**Table 3:** Concentration of lactobionic acid and lactose for culture No. 2.

Time [h]	Lactose [g/L]	Lactobionic Acid [g/L]
0	8.57 ± 0.015	0.52 ± 0.001
3	7.86 ± 0.016	0.47 ± 0.001
18	7.73 ± 0.017	0.00 ± 0
22	7.53 ± 0.016	0.76 ± 0.002
27	5.37 ± 0.014	0.74 ± 0.002
42	3.35 ± 0.012	0.00 ± 0
46	1.10 ± 0.005	0.00 ± 0
51	1.60 ± 0.007	0.00 ± 0
73	0.50 ± 0.001	0.00 ± 0
97	0.48 ± 0.001	0.00 ± 0
116	0.29 ± 0.001	0.00 ± 0

Free *Pseudomonas taetrolens* DSM 21104; culture time: 116 h.

**Table 4:** Concentration of lactobionic acid and lactose for culture No. 3.

Time [h]	Lactose [g/L]	Lactobionic Acid [g/L]
0	18.75 ± 0.03	0.00 ± 0
19	20.90 ± 0.031	0.00 ± 0
22	21.80 ± 0.031	0.00 ± 0
25	21.11± 0.032	0.00 ± 0
45	20.29 ± 0.03	0.00 ± 0
49	10.93 ± 0.01	0.70 ± 0.002
51	6.38 ± 0.007	0.73 ± 0.002
69	5.52 ± 0.006	0.00 ± 0
71	5.00 ± 0.005	0.00 ± 0
91	17.57 ± 0.007	1.36 ± 0.005
95	8.81 ± 0.035	0.94 ± 0.002
114	4.03 ± 0.004	0.00 ± 0

Free *Pseudomonas taetrolens* DSM 21104; culture time: 114 h; lactose was added at 71 h of culture, obtaining a 2% concentration in the bioreactor.

**Table 5:** Concentration of lactobionic acid and lactose for culture No. 4.

Time [h]	Lactose [g/L]	Lactobionic Acid [g/L]
0	14.97 ± 0.032	0.00 ± 0
21	14.82 ± 0.031	0.39 ± 0.001
45	9.41 ± 0.018	0.41 ± 0.001
48	5.38 ± 0.006	0.47 ± 0.002
54	3.37 ± 0.004	2.74 ± 0.006
70	2.53 ± 0.003	2.37 ± 0.006
74	1.64 ± 0.0015	1.43 ± 0.003
94	1.53 ± 0.014	0.98 ± 0.002
99	1.34 ± 0.012	0.00 ± 0
118	0.98 ± 0.001	0.00 ± 0
142	0.00 ± 0	0.00 ± 0
147	0.00 ± 0	0.00 ± 0
166	0.00 ± 0	0.00 ± 0
169	6.33 ± 0.007	0.00± 0
190	4.44 ± 0.004	0.00 ± 0
214	2.25 ± 0.002	0.82 ± 0.002
238	1.55± 0.001	0.81 ± 0.002
262	1.47 ± 0.001	0.79 ± 0.002
286	1.36 ± 0.001	0.77 ± 0.002
310	1.23 ± 0.001	0.74 ± 0.002
334	1.04 ± 0.001	0.69 ± 0.001

Microencapsulated *Pseudomonas taetrolens* 1; culture time: 334 h; fresh whey was added at 166 h.

**Table 6:** Concentration of lactobionic acid and lactose for culture No. 5.

Time [h]	Lactose [g/L]	Lactobionic Acid [g/L]
0	16.52 ± 0.034	0.46 ± 0.001
23	11.94 ± 0.021	0.40 ± 0.001
47	1.19 ± 0.002	0.34 ± 0.001
73	1.10 ± 0.002	0.00 ± 0
95	1.10 ± 0.002	0.00 ± 0
121	1.08 ± 0.002	0.00 ± 0
143	0.96 ± 0.002	0.00 ± 0
169	0.78 ± 0.001	0.00 ± 0
193	0.00 ± 0	0.00 ± 0
194	6.36 ± 0.015	0.00 ± 0
220	3.14 ± 0.007	0.00 ± 0
247	0.99 ± 0.002	0.80 ± 0.002
261	0.54 ± 0.001	0.74 ± 0.002
284	0.00 ± 0	0.00 ± 0

Free *Pseudomonas taetrolens* 4; culture time: 284 h; fresh whey was added at 193 h.

**Table 7:** Concentration of lactobionic acid and lactose for culture No. 6.

Time [h]	Lactose [g/L]	Lactobionic Acid [g/L]
0	18.46 ± 0.031	0.00 ± 0
28	8.60 ± 0.016	0.98 ± 0.002
44	8.95 ± 0.016	1.50 ± 0.003
67	5.57 ± 0.005	1.39 ± 0.003
95	4.29 ± 0.004	0.70 ± 0.001
118	4.23 ± 0.004	0.68 ± 0.001
141	4.54 ± 0.004	0.55 ± 0.001
167	3.66 ± 0.003	0.41 ± 0.001
187	3.55 ± 0.002	0.38 ± 0.001
210	3.34 ± 0.007	0.31 ± 0.001
231	3.10 ± 0.007	0.21 ± 0.001
252	12.24 ± 0.004	0.15 ± 0.001
275	12.20 ± 0.004	0.00 ± 0
302	12.12 ± 0.004	0.00 ± 0
232	11.69 ± 0.003	0.00 ± 0
347	11.54 ± 0.003	0.00 ± 0
378	11.22 ± 0.002	0.00 ± 0
396	11.26 ± 0.002	0.00 ± 0
421	11.16 ± 0.002	0.00 ± 0

Microencapsulated *Pseudomonas taetrolens* 4; culture time: 421 h; fresh whey was added at 231 h.

**Table 8:** Concentration of lactobionic acid and lactose for culture No. 7.

Time [h]	Lactose [g/L]	Lactobionic Acid [g/L]
0	24.85 ± 0.068	0.70 ± 0.001
24	25.11 ± 0.070	1.72 ± 0.003
44	24.76 ± 0.067	1.61 ± 0.003
65	23.99 ± 0.065	0.95 ± 0.001
89	23.93 ± 0.065	0.51 ± 0.001
113	20.68 ± 0.051	0.00 ± 0
138	20.93 ± 0.052	0.00± 0

Free *Pseudomonas taetrolens* 1; culture time: 138 h.

**Table 9:** Concentration of lactobionic acid and lactose in culture 8.

Time [h]	Lactose [g/L]	Lactobionic Acid [g/L]
0	39.90 ± 0.1	0.00 ± 0
8	45.18 ± 0.110	1.32 ± 0.002
25	39.79 ± 0.1	6.75 ± 0.005
54	24.85 ± 0.02	28.66 ± 0.01
80	23.87 ± 0.019	34.54 ± 0.02
102	21.22 ± 0.017	37.42 ± 0.03

Free *Pseudomonas taetrolens* 4′; culture time: 102 h; the concentration of added lactose in the bioreactor was 2% from the very beginning.

**Table 10:** Concentration of lactobionic acid and lactose in culture 9.

Time [h]	Lactose [g/L]	Lactobionic Acid [g/L]
0	44.68 ± 0.12	0.79 ± 0.001
21	41.59 ± 0.105	1.43 ± 0.002
45	32.22 ± 0.061	1.35 ± 0.002
71	25.60 ± 0.032	1.23 ± 0.002
93	24.67 ± 0.031	1.23 ± 0.002
117	22.39 ± 0.028	1.22 ± 0.002
141	15.63± 0.030	1.98 ± 0.003
166	18.40 ± 0.033	1.17 ± 0.002

Microencapsulated *Pseudomonas taetrolens* 4′; culture time: 166 h; the concentration of added lactose in the bioreactor was 2% from the very beginning.

**Table 11:** Results of metabolically active cells during culture in the bioreactor.

SAMPLES	DEAD	SD/2	MID-ACTIVE_1	SD/2	MID-ACTIVE_2	SD/2	ACTIVE	SD/2
1 day	14.3	0.1	81.4	0.1	0.5	0.0	0.0	0.0
1 day (2nd sample)	19.1	0.2	76.5	0.5	1.2	0.1	0.1	0.0
2 day	79.4	0.3	8.3	0.1	7.1	0.3	0.3	0.0
3 day	64.4	0.2	28.7	0.1	1.3	0.2	0.1	0.0
4 day	54.5	0.3	30.7	0.3	8.1	0.0	3.5	0.3
5 day	56.9	0.3	35.4	0.3	5.6	0.1	0.4	0.0
6 day	39.4	0.2	46.9	0.1	7.8	0.1	3.0	0.0
7 day	36.5	0.1	53.7	0.3	5.3	0.1	1.2	0.0
8 day	18.4	0.1	47.4	0.5	23.5	0.8	7.0	0.3
9 day	16.7	0.2	33.5	0.1	25.6	0.4	20.1	0.6
10 day	10.5	0.0	45.8	0.3	16.6	0.3	14.0	0.3
11 day	6.0	0.1	14.1	0.1	30.1	0.5	39.0	0.4
12 day	23.4	0.4	22.8	0.3	23.9	0.6	20.5	0.6
13 day	15.2	0.2	28.3	0.2	13.5	0.2	32.3	0.5
14 day	19.9	0.0	25.4	0.4	19.5	0.1	25.0	0.3
15 day	19.0	0.6	50.0	0.3	7.4	0.2	10.5	0.2
16 day	26.1	0.3	31.9	0.7	18.4	0.1	12.0	0.2
17 day	22.9	0.4	28.6	0.4	15.2	0.2	23.2	0.6
17 day (2nd sample)	35.7	0.3	21.5	0.2	22.8	0.4	14.3	0.4
18 day	24.2	0.2	27.4	0.2	18.7	0.4	19.7	0.4
19 day	24.7	0.2	27.5	0.3	19.6	0.3	18.9	0.2

**Table 12:** The number of viable cells of microorganisms encapsulated in microcapsules before and after the digestion process.

Strain	Microcapsules Before Digestion [cfu/g]	Broth Medium After Stomach [cfu/mL]	Broth Medium After Small Intestine [cfu/mL]	Microcapsules After Digestion [cfu/g]
*B. bifidum* DSM 20239	2.2 × 10^9^	1.0 × 10^3^	2.0 × 10^4^	7.0 × 10^4^
*B. bifidum* DSM 20082	2.08 × 10^8^	4.0 × 10^2^	1.9 × 10^6^	6.5 × 10^6^
*B. bifidum* DSM 20215	1.9 × 10^9^	2.5 × 10^2^	3.0 × 10^2^	7.6 × 10^4^
*B. bifidum* DSM 20456	2.8 × 10^8^	4.25 × 10^1^	3.75 × 10^1^	2.0 × 10^5^

**Table 13:** Concentration of lactobionic acid in microcapsules for individual strains of probiotic bacteria.

Strain	Concentration of Lactobionic Acid [%]
*Bifidobacterium bifidum* DSM 20239	2%
*Bifidobacterium bifidum* DSM 20082	2%
*Bifidobacterium bifidum* DSM 20215	2%
*Bifidobacterium bifidum* DSM 20456	2%

0.1% (*w*/*v*) is equivalent to 1 mg/mL LAB.

## Data Availability

Data are contained within the article.
